# Evaluation of cultivated land quality using attention mechanism-back propagation neural network

**DOI:** 10.7717/peerj-cs.948

**Published:** 2022-04-11

**Authors:** Yulin Liu, Jiaolong Li, Chuang Liu, Jiangshu Wei

**Affiliations:** College of Information Engineering, Sichuan Agricultural University, Ya’an, Sichuan, China

**Keywords:** Attention Mechanism, BP neural network, Cultivated land quality, Deep learning

## Abstract

Cultivated land quality is related to the quality and safety of agricultural products and to ecological safety. Therefore, reasonably evaluating the quality of land, which is helpful in identifying its benefits, is crucial. However, most studies have used traditional methods to estimate cultivated land quality, and there is little research on using deep learning for this purpose. Using Ya’an cultivated land as the research object, this study constructs an evaluation system for cultivated land quality based on seven aspects, including soil organic matter and soil texture. An attention mechanism (AM) is introduced into a back propagation (BP) neural network model. Therefore, an AM-BP neural network that is suitable for Ya’an cultivated land is designed. The sample is divided into training and test sets by a ratio of 7:3. We can output the evaluation results of cultivated land quality through experiments. Furthermore, they can be visualized through a pie chart. The experimental results indicate that the model effect of the AM-BP neural network is better than that of the BP neural network. That is, the mean square error is reduced by approximately 0.0019 and the determination coefficient is increased by approximately 0.005. In addition, this study obtains better results via the ensemble model. The quality of cultivated land in Yucheng District is generally good, i.e.,mostly third and fourth grades. It conforms to the normal distribution. Lastly, the method has certain to evaluate cultivated land quality, providing a reference for future cultivated land quality evaluation.

## Introduction

Cultivated land quality represents comprehensive properties of soil and lays the foundation for soil as a part of terrestrial ecology and crop production (Smith et al., 2014). Quality of arable land affects food production. Due to rapid socio-economic development and urbanization, the amount of cultivated land in China has been decreasing (Lin et al., 2022). In the case of Ya’an City, the situation is not optimistic. Its land carrying capacity faces increasing challenges as industrialisation and urbanisation have become the major themes of China’s economic development (Ding & Peng, 2018). In addition, cultivated land, which is affected by various factors, including the use of new technologies and new varieties, face potential safety hazards, such as chemical and biological pollution, which damage long-term economic benefits and environmental protection (Sun et al., 2018). Therefore, conducting farmland quality evaluation research is imperative to ensure food security and support new development.

In the evaluation of cultivated land quality, evaluation methods vary with different indicators selected under local conditions because cultivated land in different regions has distinct properties. With the updating of computer technology and mathematical knowledge, methods for farmland quality evaluation are developing rapidly (Liu, Zhang & Guo, 2010; Xie et al., 2018). Different methods may affect the evaluation result due to the selection of evaluation indicators, the division of indicator levels, or the weighted method (Viana et al., 2021). Therefore, formulating a reasonable cultivated land evaluation system that is conducive to conducting land development research is necessary. At present, common methods used to evaluate the quality of cultivated land include distance, number axis, factor, and sample plot methods (Tan et al., 2020). However, these methods cannot ensure the objectivity of evaluation because of the inclusion of subjective factors. Therefore, researchers are still exploring index systems for cultivated land quality. With the continued development of science and technology, deep learning algorithms, such as attention mechanism (Vaswani et al., 2017) (AM) and meta-learning (Finn, Abbeel & Levine, 2017), have emerged. Most of these algorithms are used in target detection and recognition. Some of these models have also been improved and applied to regression related to agriculture and agricultural produce, yielding good results (Nassar et al., 2020; Zhu et al., 2020; Gao et al., 2021). These algorithms can exhibit a combination of advantages by learning from one another and through various means of integration. In the era of information digitisation, information dominance constitutes a basic feature of the future; thus, studying how new methods can be combined with the evaluation of cultivated land quality is necessary (Liu et al., 2020b). Some scholars have achieved good results in their endeavour to integrate a back propagation (BP) neural network (Hao et al., 2021), a convolutional neural network (Liu et al., 2020a), a genetic algorithm (Liu et al., 2019), and the particle swarm optimisation algorithm (Wang et al., 2020) into the evaluation of cultivated land quality. However, the existing study of cultivated land quality is still lacking in combination with new deep learning algorithms.

A BP neural network is a multilayered feedforward neural network, which has strong nonlinear mapping ability and simple structure (Hu et al., 2021). It can build a model based on the existing data and use this model to solve regression or classification problems. Considering its extensive applications, a BP neural network constitutes the basis of the current study. AM is conducive to focusing attention on the key points in a neural network whilst disregarding unimportant factors (Pabón et al., 2022). Therefore, it can reduce the computational burden of processing high-dimensional input data and decrease data dimension through a structurally selected input subset.

The current study aims to find a more accurate evaluation method that is in line with reality and then develop it into a more standardised and scientific direction whilst providing a reference for other researchers. This work selects the cultivated land in the Yucheng District of Ya’an as the research object, devises a scientific system of cultivated land evaluation indicators, and evaluates the quality of cultivated land based on the AM-BP model, which can provide the attention score. In addition, an ensemble model based on AM-BP is used to reduce errors. Based on the model results, a map is drawn to illustrate the condition of cultivated land quality in the Yucheng District of Ya’an City. This will help the local government to understand the status of cultivated land in this region, promoting the structural adjustment of agriculture. Moreover, it is conducive to the sustainable development.

## Materials and Methods

### Overview of the research area

Located in the transition zone between the western edge of the Sichuan Basin and the Qinghai–Tibet Plateau, Yucheng District covers an area of 1067.3 km^2^, and it belongs to one of the mountain counties around the basin. With the impact of urbanisation, the amount of arable land resources in China is decreasing (Deng et al., 2015), with the per capita arable land area only 2/3 that of the world’s average. Soil quality is seriously degraded whilst indiscriminate and excessive cultivation and occupation for construction occur ubiquitously. Therefore, the use of artificial intelligence methods for evaluating arable land is indispensable to understanding the current status of the quantity and quality of arable land resources in local areas. On the basis of this assessment, targeted arable land protection and management projects can be implemented to achieve smart and sustainable agricultural development.

### Data source

The research data are derived from soil testing and fertilisation in Yucheng District, and they are provided by the Yucheng District Agricultural Bureau of Ya’an City. The average area of each sampling unit is 100–200 mu, setting up representative sampling points. The sampling depth is generally 0–20 cm, and the orchard can be collected in layers of 0 to 40 cm or 0–60 cm.

The accuracy rate is controlled by the use of standard samples. To identify the error, we add one standard sample for each batch. When the difference between the testing result and the standard value is out of the range of the standard deviation, the experimental procedure should be checked to find out the reason.

### Data preprocessing

Data preprocessing is a significant task. Different dimensional units affect the results of our numerical analysis. Therefore, data must be standardised to ensure their comparability and eliminate such effects. The min-max standardisation for the data is as follows:

(1) \begin{eqnarray*}Z= \frac{x-{x}_{\mathrm{min}}}{{x}_{\mathrm{max}}-{x}_{\mathrm{min}}} ,\end{eqnarray*}

where *x*_max_ is the maximum value of the sample data, and *x*_min_ is the minimum value of the sample data.

### Construction of an index system

In accordance with the regional characteristics of cultivated land in the Yucheng District of Ya’an City, combined with data access, data statistics, expert consultation, and other methods, indexes, such as surface soil texture, soil organic matter, soil nutrient elements, and pH value, are selected from natural elements to build a quality evaluation system for Ya’an cultivated land, as presented in Table 1.

**Table 1:** Cultivated land quality evaluation index system in Yucheng District.

**Factor**		**Level 1**		**Level 2**		**Level 3**		**Level 4**		**Level 5**		**Level 6**	
		**Index value**	**Score**	**Index value**	**Score**	**Index value**	**Score**	**Index value**	**Score**	**Index value**	**Score**	**Index value**	**Score**
Natural factor	Surface soil texture	Loam	6	Clay	4	Sand	2	Gravelly Soil	1			
	Soil organic matter	≥40	6	[30, 40)	5	[20, 30)	4	[10, 20)	3	[6, 10)	2	<6	1
	pH value	[6.0, 7.9)	6	[5.5, 6.0) or [7.9, 8.5)	5	[5.0, 5.5) or [8.5, 9.0)	4	[4.5, 5.0)	3	<4.5 or ≥9.0	2		
	Available P/ppm	≥40	6	[20, 40)	5	[10, 20)	4	[5, 10)	3	[3, 5)	2	<3	1
	Total nitrogen%	≥2	6	[1.5, 2)	5	[1.0, 1.5)	4	[0.75, 1)	3	[0.5, 0.75)	2	<0.5	1
	Available K/ppm	≥200	6	[150, 200)	5	[100, 150)	4	[50, 100)	3	[30, 50)	2	<30	1
	Available N/ppm	≥150	6	[120, 150)	5	[90, 120)	4	[60, 90)	3	[30, 60)	2	<30	1

### Model building

### BP neural network

A BP neural network is composed of an input layer, a hidden layer, and an output layer. In the current study, the number of input layer nodes is seven due to the cultivated land characteristics of the data source and the obtained score. Meanwhile, the output is 1, which represents the quality score of cultivated land. When the number of neurons in the hidden layer is suitable, better results can be obtained (Karsoliya, 2012). The selection of the number of hidden layer nodes is presented in Eq. (2):

(2) \begin{eqnarray*}n=\sqrt{a+b}+c,\end{eqnarray*}

where *n*, *a*, and *b* represent the numbers of hidden layer nodes, input layer nodes, and output layer nodes, respectively; and *c* represents an integer between 1 and 10.

Therefore, the mean square error (MSE) of the model results at different numbers of hidden layer nodes is adopted, as shown in Table 2.

**Table 2:** The training times and errors of BP neural networks with different hidden layer nodes.

**The number of hidden layer node**	**Training times**	**MSE**
3	30	0.1348
4	30	0.1596
5	30	0.0926
6	30	0.0976
7	30	0.1855
8	30	0.1171
9	30	0.0996
10	30	0.0961
11	30	0.1514
12	30	0.1105

**Figure 1:**
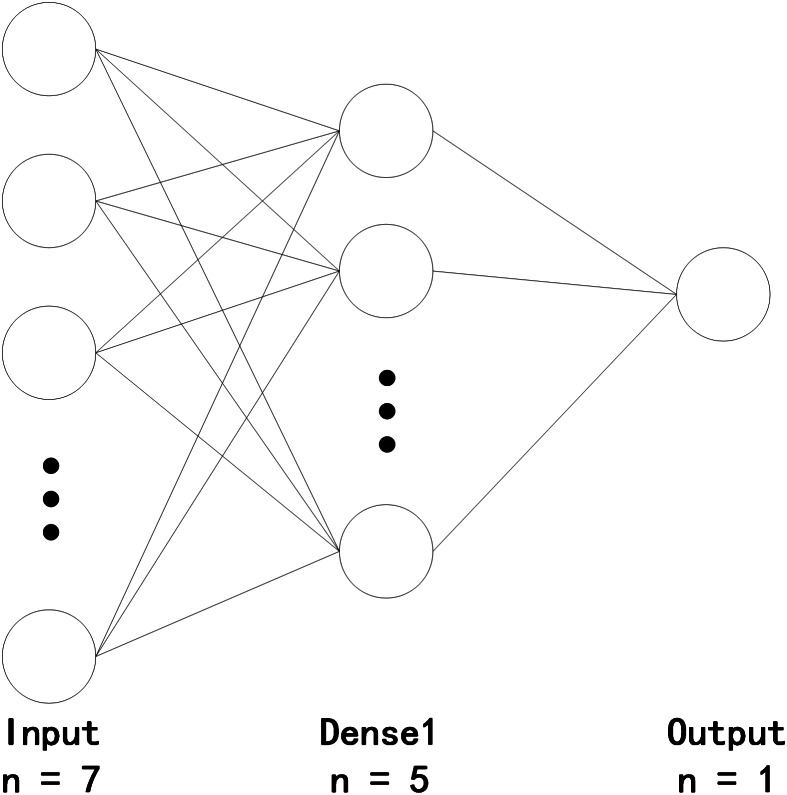
Quality evaluation of cultivated land based on the BP neural network.

As indicated by the result, the effect of the model is the best when the number of hidden layers is five. In the BP model, the optimiser used is Adam, the learning rate is 0.01, the maximum training time is 500, and batch size is 100. Thus, the establishment of the BP neural network model is completed, as illustrated in Fig. 1.

**Figure 2:**
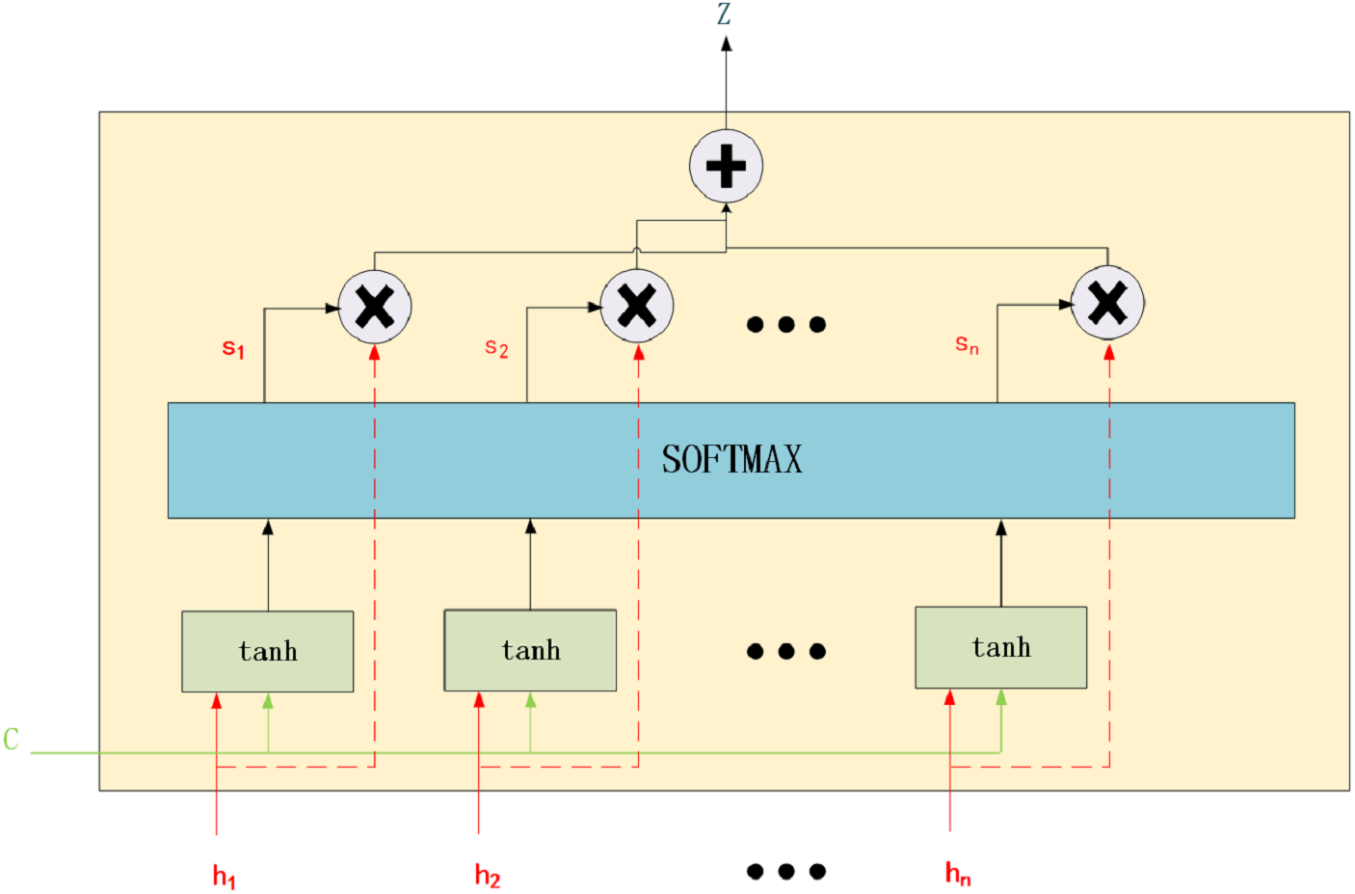
Attention mechanism.

### AM

Attention is one of the most important components of neural networks that are adept at understanding sequences, including video sequences, action sequences in real life, or a sequence of inputs, such as voice, text, or any other data. Obtaining a reasonable vector representation is typically difficult because the input sequence of long short-term memory or a recurrent neural network may be too long. Thus, AM is created. The attention mechanism not only greatly improves the efficiency and accuracy of perceptual information processing, but also provides the interpretability for the model generation process (Niu, Zhong & Yu, 2021). At present, the application of AM to the field of agriculture is gradually becoming popular (Zhao et al., 2021; Zhao, Huang & Nie, 2021). However, it has no relevant application to cultivated land quality.

AM was proposed by Bahdanau, Cho & Bengio (2014). Its original purpose was to automatically search relevant parts from the input sequence of the current predictor by constructing a mechanism. These authors used AM to build a better translation model, proving that AM is reasonable.

To understand AM, we can divide it into three steps: score function, alignment function, and generate context vector function.

Score Function: The similarity between the environment vector and the current input vector is measured. The important input information that should be focused on in the current environment is identified. These authors used AM to build a better translation model, proving that AM is reasonable. The structure of AM is shown in Fig. 2. The calculation method is presented in Eq. (3):

(3) \begin{eqnarray*}{e}_{ij}=a \left( c,{h}_{i} \right) ={v}_{a}^{T}\tanh \nolimits \left( {W}_{a}\ast c+{U}_{a}\ast {h}_{i} \right) ,\end{eqnarray*}

where *e*_*ij*_ is the alignment model that is used to measure the alignment of the *j*-th word at the input position to the *i*-th word at the output position. *a* and *c* represent the alignment model function and the context vector. *h*_*i*_ is the input. *v*_*a*_, *W*_*a*_ and *U*_*a*_ are the weight matrices.

Alignment Function: Attention weight is calculated. Softmax is typically used for normalisation. The calculation method is presented in Eq. (4):

(4) \begin{eqnarray*}{\alpha }_{ij}= \frac{\exp \nolimits \left( {e}_{ij} \right) }{\sum _{k=1}^{{T}_{x}}\exp \nolimits \left( {e}_{ik} \right) } ,\end{eqnarray*}

where *T*_*x*_ represents the last symbol of the input vector.

Generate Context Vector Function: The output vector is obtained with the attention weight. The calculation method is presented in Eq. (5):

(5) \begin{eqnarray*}{z}_{ij}=\sum _{i}{\alpha }_{ij}\ast {h}_{i}.\end{eqnarray*}

### Ensemble model

The ensemble model imports the data trains multiple models and integrates one model by a certain method. It is easy to understand, simple and efficient. This method is one of the common approaches for improving the model effect. The methods for the ensemble model are generally divided into voting, averaging, boosting, and stacking. In the current study, we use the averaging method to obtain the result, as shown in Eq. (6):

(6) \begin{eqnarray*}\mathrm{result}=\sum _{t=1}^{n}{\mathrm{Weight}}_{i}\ast {P}_{i},\end{eqnarray*}

where *n*, Weight_*i*_ and *P*_*i*_ represent the number of models, model weight, and the predicted value of the model, respectively.

### Evaluation index

To evaluate the consistency between predicted and true values, MSE and the determination coefficient (*R*^2^) were used to assess the performance of the models in this study, as shown in Eqs. (7) and (8):

(7) \begin{eqnarray*}& & \mathrm{MSE}= \frac{1}{n} \sum _{i=1}^{n}{ \left( {\hat {y}}_{i}-{y}_{i} \right) }^{2},\end{eqnarray*}

(8) \begin{eqnarray*}& & {R}^{2}= \frac{\sum _{i=1}^{n}{ \left( {y}_{i}-{\overline{y}}_{i} \right) }^{2}}{\sum _{i=1}^{n}{ \left( {\hat {y}}_{i}-{\overline{y}}_{i} \right) }^{2}} ,\end{eqnarray*}

where *n* is the total number of samples, and ${\hat {y}}_{i}$, *y*_*i*_ and ${\overline{y}}_{i}$ represent the predicted values of *i*-th sample, the true values of the *i*-th sample and the average values of the total samples.

### Laboratory environment

This research is completed using a Windows 10 operating system and a TensorFlow 2.0 framework. The experimental hardware environment is an HP8574 motherboard. The CPU model is Intel Core i7 9750H. The GPU model is NVIDIA GeForce GTX 1660 Ti 6G. The software environment is CUDA 10.0, CUDNN 7.6.5, and Python 3.7.

## Results

### Results of different machine learning methods

**Figure 3:**
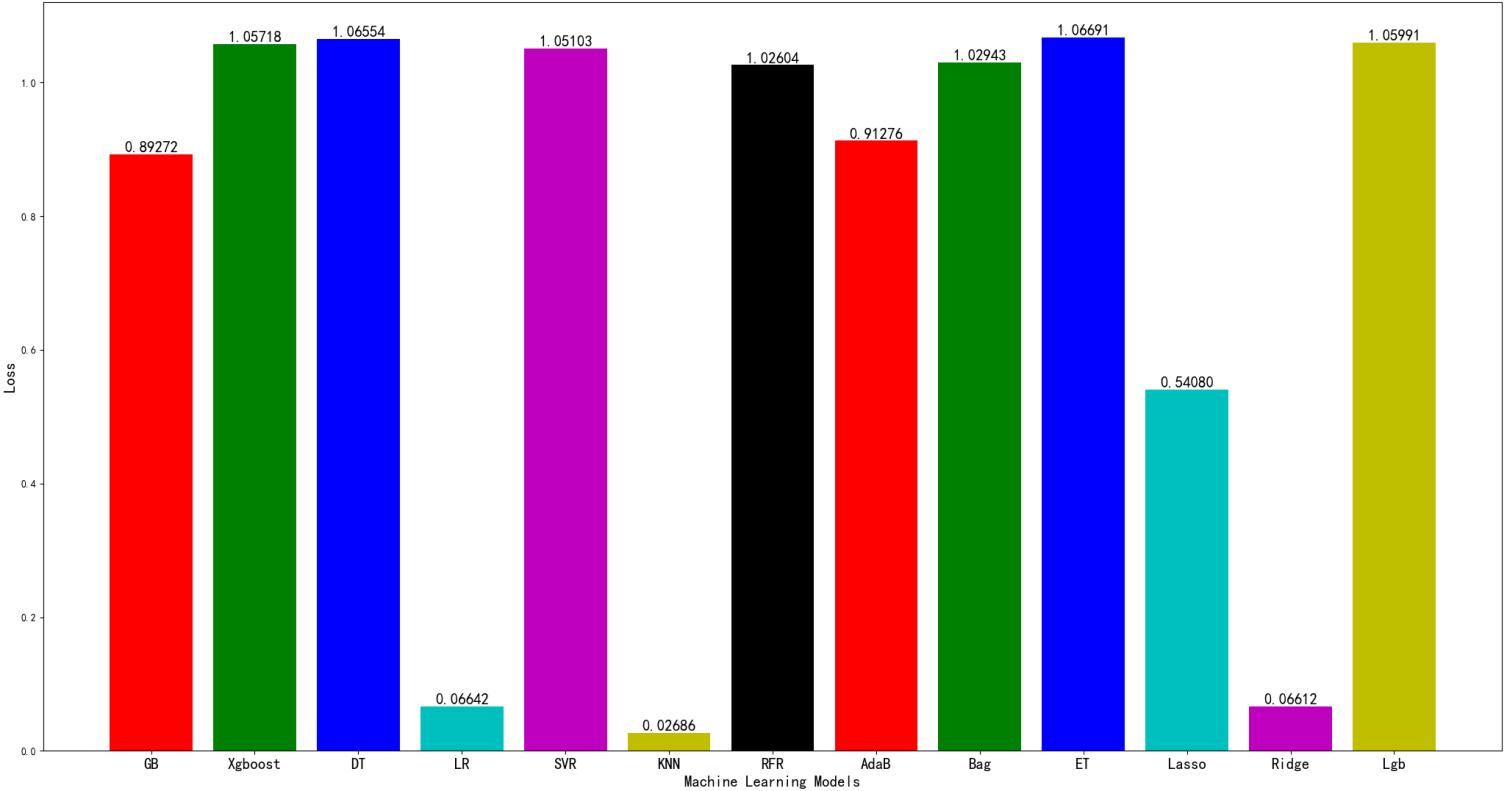
Performance of different machine learning models on testing set.

Machine learning has five characteristics: accuracy, automation, speed, customisation and scale. It is applied in our daily lives. To test the effect of the machine learning model on farmland datasets and the subsequent ensemble model, 2150 samples from the datasets are used to train 13 machine learning algorithm models, whilst 920 samples are used for testing. The 13 algorithms are as follows: a gradient boosting, extreme gradient boosting (XGBoost), decision tree, linear regression, support vector regression (SVR), *k*-nearest neighbour (*k*-NN), random forest regression, adaptive boosting (AdaBoost), bootstrap aggregating, extreme tree regression, lasso regression, ridge regression and light gradient boosting machine (LightGBM). They respectively correspond to GB, Xgboost, DT, LR, SVR, *k*-NN, RFR, AdaB, Bag, ET, Lasso, Ridge, and Lgb in Fig. 3. The MSE of each model on the test set is obtained. The result is presented in Fig. 3.

As shown in Fig. 3, the *k*-NN model exhibits the best effect and the minimum MSE. It is more favourable for the subsequent model fusion to reduce MSE. It is selected as one of the subsequent model fusion algorithms.

### Comparison of model effects

**Figure 4:**
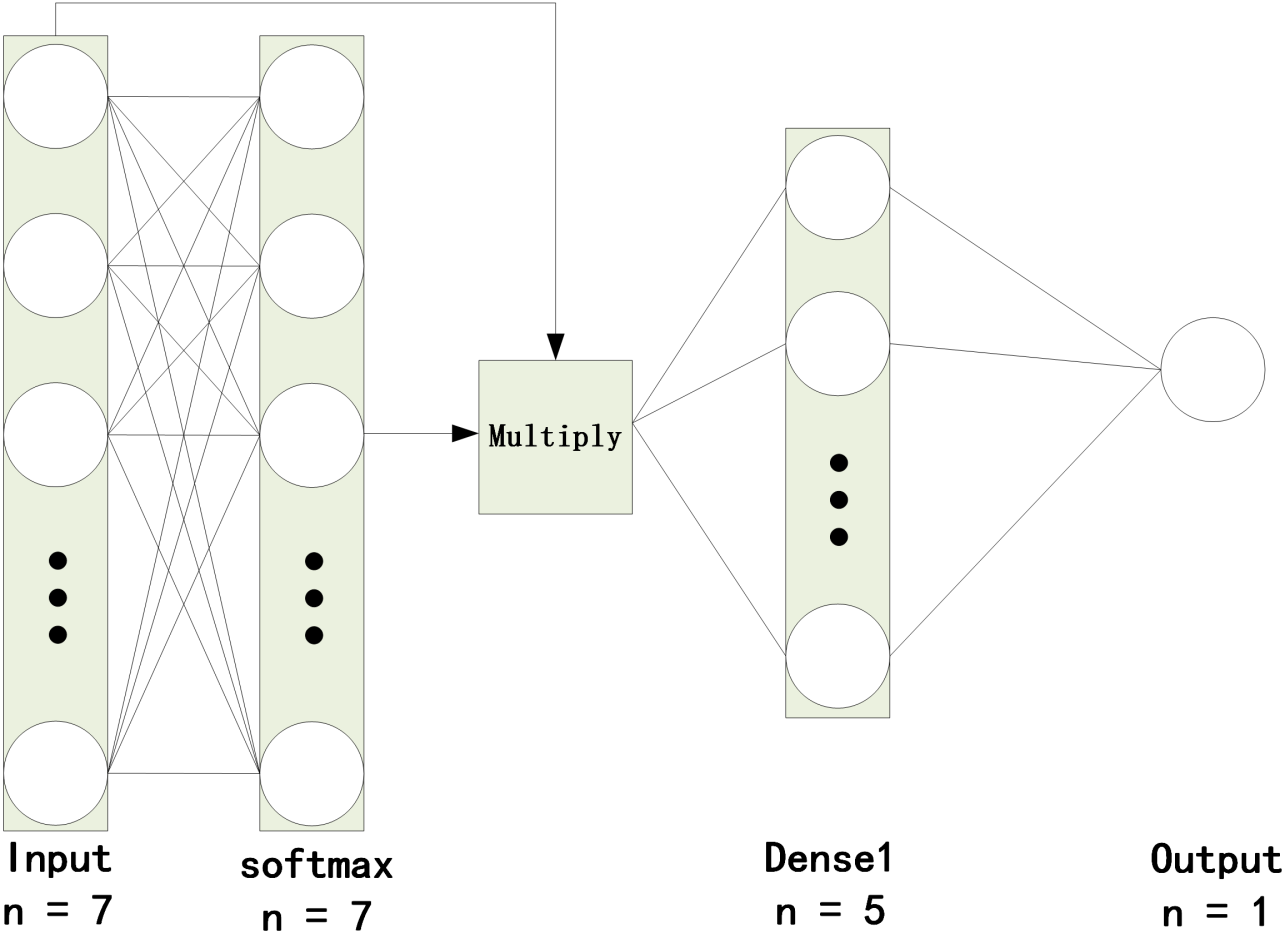
Quality evaluation of cultivated land based on the AM-BP neural network.

To integrate AM into the model, we combine the model with the characteristics of AM, as shown in Fig. 4. In the current study, the attention layer is designed to have two layers: a softmax layer and a multiply operation. The softmax layer, which is added after the input layer, can obtain attention scores and then multiply the scores by the input.

**Figure 5:**
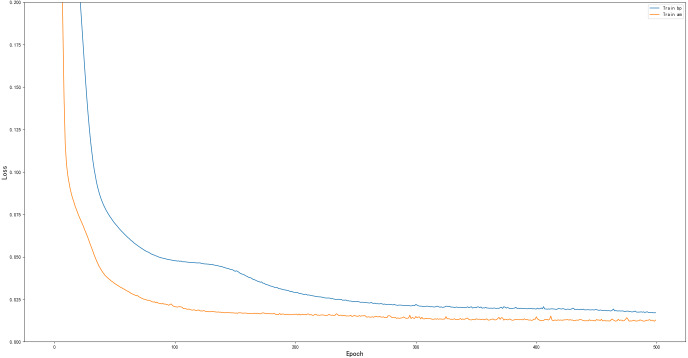
Performance comparison of BP and AM-BP on the training set.

As a common practice in machine learning, data are divided into 2150 training samples and 920 test samples. The other parameters are the same except for the different network structures. These parameters are applied to the BP and AM-BP models. The results are presented in Fig. 5. The AM-BP model is better than the BP model. The MSE is reduced by approximately 0.002 (approximately 12%), the *R*^2^ is increased by approximately 0.005 at the same time.

The MSEs of the BP and AM-BP models on the test set is provided in Table 3. They show that the model with AM is more sensitive to data and exhibits a better learning effect. The AM-BP model also has shortcomings. It allocates attention through weights, which increases the complexity of the network. In addition, the sequential relationship in input cannot be learned by the model.

**Figure 6:**
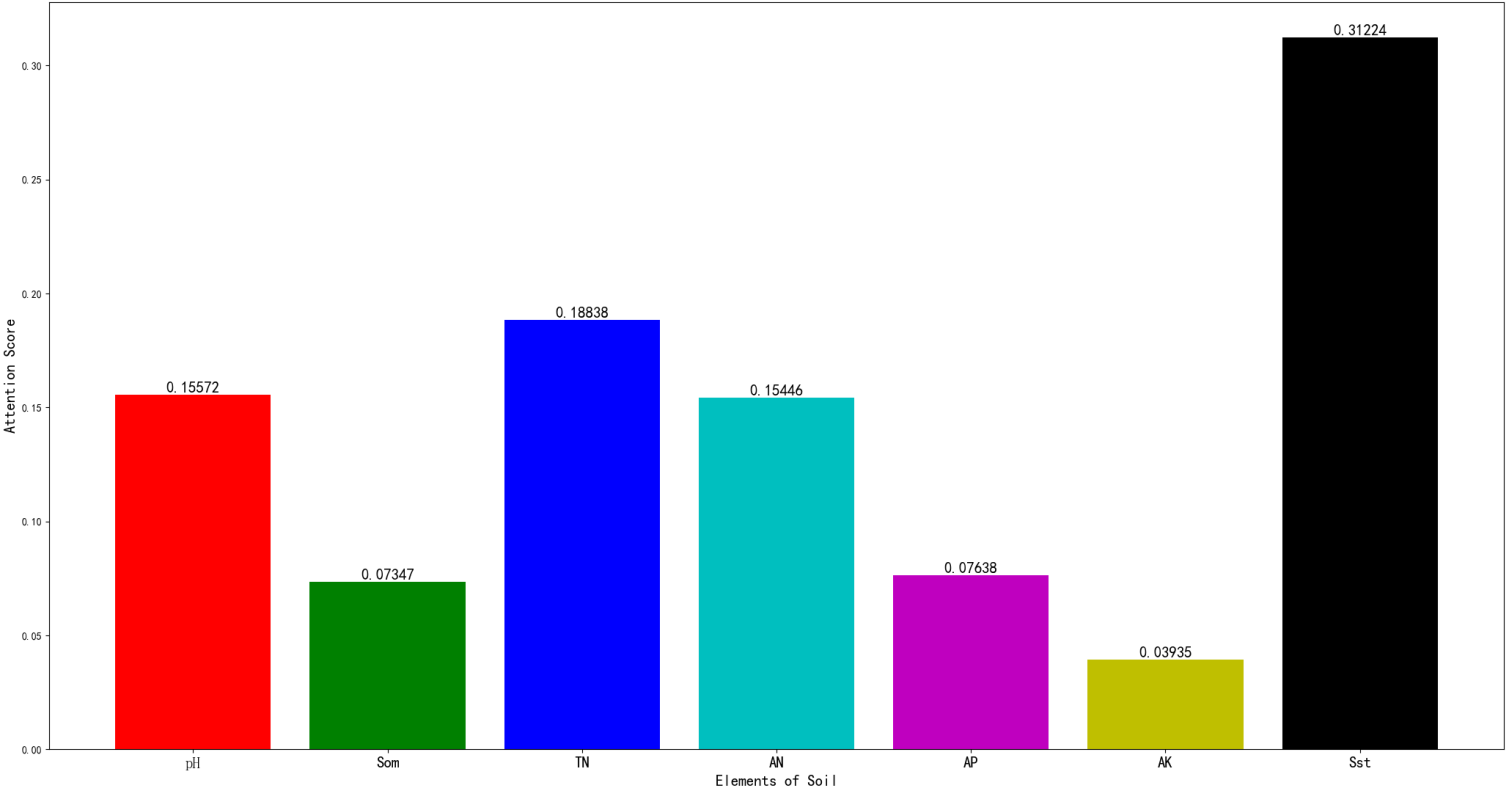
The results of attention score.

The AM-BP model can return the value of the attention score, as shown in Fig. 6.

In this figure, pH, SST, TN, AN, AP, AK, and SOM correspond to pH value, surface soil texture, total nitrogen, available nitrogen, available phosphorus, available potassium, and soil organic matter, respectively.

When the score is higher, the correlation between the model and related items is higher. As shown in Fig. 6, soil texture and alkali-hydrolysable nitrogen exert more important influences on the score. Soil texture directly affects the physical and chemical properties and fertility status of the soil. For example, loam soil exhibits better cultivability and fertility, and its capability to supply and maintain fertilizer is moderate. It is most suitable for the growth of fruit trees and vegetables. The cultivability and fertility of clay are worse than those of loam. Large pores are lacking between soil particles, and air and water permeability are poor. Clay is more suitable for planting rice.

### Effects of ensemble model

**Figure 7:**
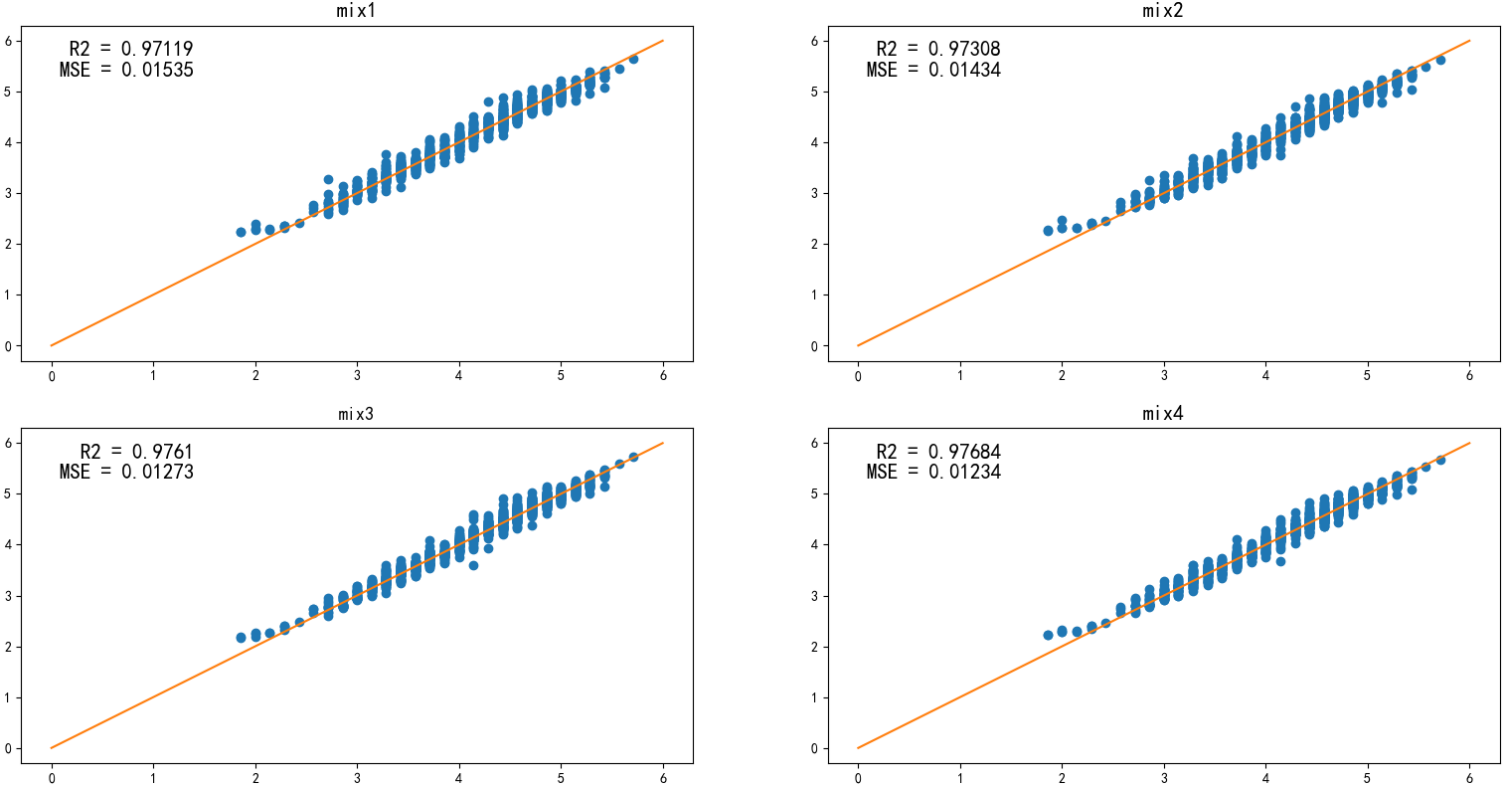
The effect of the ensemble model.

In general, when the learner’s appointment is more accurate and diversified, its integration is better. To improve the accuracy of the model further, the *k*-NN, BP, and AM-BP models are fused, and the result is presented in Fig. 7. mix1, mix2, mix3, and mix4 are the results of the fusion of the *k*-NN, BP, and AM-BP models with certain weights, as shown in Table 4. In addition to the mix1 and mix2 model, the accuracy of the two other models is significantly improved.

**Table 3:** Performance comparison of BP and AM-BP on the testing set.

**Model**	**MSE**	**R2**
BP	0.0162	0.9683
AM-BP	0.0143	0.9726

## Discussion

**Figure 8:**
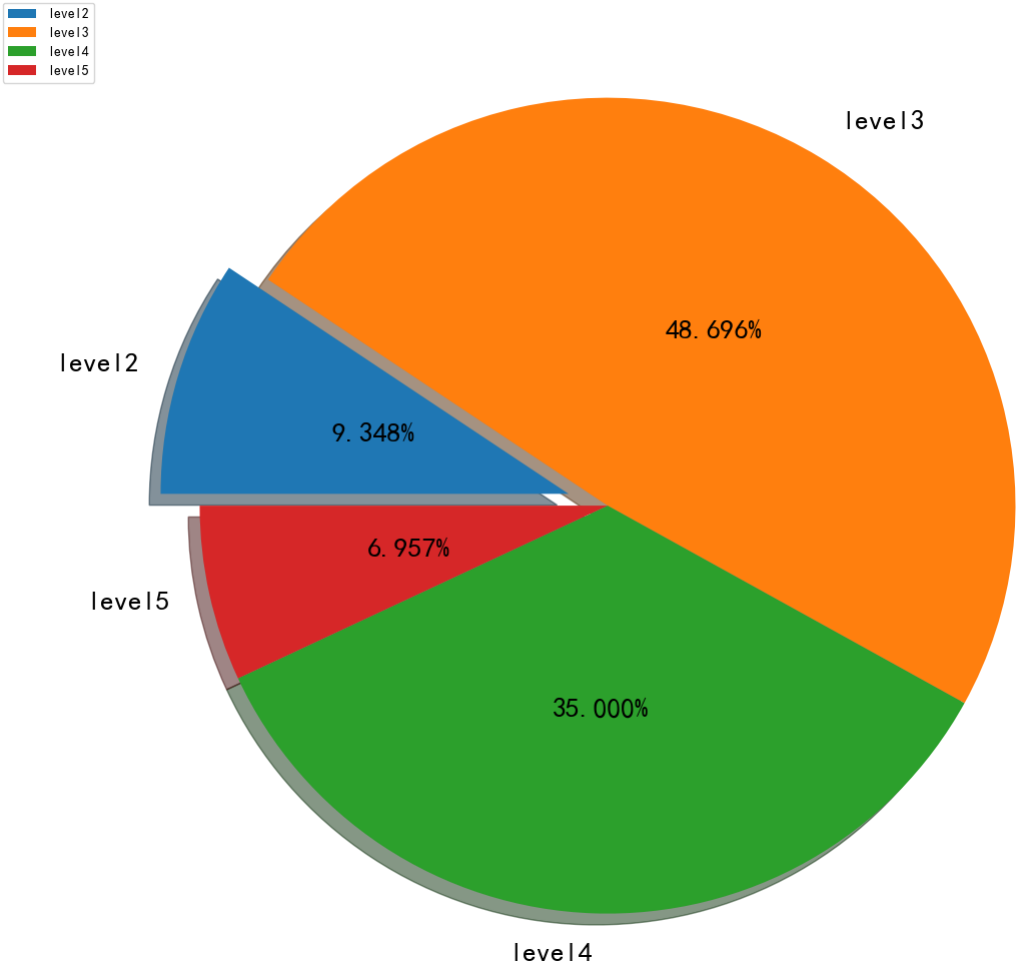
The results of Arable land quality rating.

The result of the cultivated land quality rating obtained using the AM-BP model is presented in Fig. 8. In this figure, the arable land in Yucheng District is divided into four levels. Among which, the third and fourth classes are large, accounting for approximately 80%, followed by the second class. With an extensive area of medium- and low-yield fields, the arable land in Yucheng District exhibits low quality and should be improved. To effectively protect cultivated land resources, improve the quality of cultivated land, and ensure the sustainable development of agriculture, supervision and management should be strengthened, the quality monitoring and early warning system of cultivated land should be improved and the quantity and quality information of land should be updated regularly to capture changes in fertility accurately and perform better in the allocation and improvement of arable land resources. Relevant departments can develop the quality of cultivated land by providing technical guidance and services to farmers, whilst relying on campaigns, such as soil testing and formula fertilisation, and the transformation of medium- and low-yield soil, to adopt measures to local conditions and rational farming, and thus, realise the sustainable development and utilisation of cultivated land resources.

**Table 4:** Types of ensemble model and the weights of the models.

**Model names**	**BP**	**AM-BP**	**KNN**
Mix 1	0.6	–	0.4
Mix 2	–	0.6	0.4
Mix 3	0.4	0.6	–
Mix 4	0.2	0.6	0.2

In this study, the model error is reduced and performance is improved by fusing the models. The ensemble model based on the AM-BP model obtained in this study is better than the traditional method, and MSE is significantly reduced. Although this method exhibits good performance, it still has shortcomings. The effect of the ensemble model is not evident compared with that of the AM-BP model. The subsequent research can further improve the result of the model through network pruning and knowledge distillation.

Moreover, the initial parameters of the AM-BP model are zero. It may affect the model’s accuracy. Therefore, we will add parameter optimization algorithms to improve the efficiency and stability of the AM-BP model in the next stage of the study.

## Conclusions

To determine the cultivated land quality rating in the Yucheng District of Ya’an City, a method based on a BP neural network is proposed in the current study. Combined with the situation of data, there are many experimental studies performed for this study, adjusting the structure and parameters of the network. The conclusions are as follows: (1) The AM-BP model with MSE of 0.0143 were more reliable than machine learning models. (2) The accuracy of the ensemble model significantly improved, further implying that the AM-BP model could be applied to accurately mapping cultivated land quality at the regional scale in the future. (3) The quality of cultivated land in Yucheng District is generally good, *i.e.,* mostly third and fourth grades. It conforms to the normal distribution. The land needed to be improved.

## Supplemental Information

10.7717/peerj-cs.948/supp-1 File S1 Raw data All the tested soil information in Yucheng District. These information was used to evaluate the quality of cultivated land.

10.7717/peerj-cs.948/supp-2 File S2 The experiment code ml.py, am2.py, BP.py, and Datacleaning2.py represent machine learning experiment, attention mechanism experiment, BP neural network and ensemble model experiment, data preprocessing respectively.

## Additional Information and Declarations

### Data Availability

The following information was supplied regarding data availability: The raw data and code are available in the Supplementary Files.
